# Effects of Pest Management Practices on Soil Nematode Abundance, Diversity, Metabolic Footprint and Community Composition Under Paddy Rice Fields

**DOI:** 10.3389/fpls.2020.00088

**Published:** 2020-02-19

**Authors:** Bing Yang, Qunying Chen, Xianghui Liu, Fajun Chen, Yuyong Liang, Wei Qiang, Lulu He, Feng Ge

**Affiliations:** ^1^Key Laboratory of Mountain Ecological Restoration and Bioresource Utilization & Ecological Restoration, Biodiversity Conservation Key Laboratory of Sichuan Province, Chengdu Institute of Biology, Chinese Academy of Sciences, Chengdu, China; ^2^State Key Laboratory of Integrated Management of Pest Insects and Rodents, Institute of Zoology, Chinese Academy of Sciences, Beijing, China; ^3^College of Plant Protection, Nanjing Agricultural University, Nanjing, China; ^4^Institute of Plant Protection, Jiangxi Academy of Agricultural Sciences, Nanchang, China; ^5^College of Life Sciences, University of Chinese Academy of Sciences, Beijing, China; ^6^CAS Center for Excellence in Biotic Interactions, University of Chinese Academy of Sciences, Beijing, China

**Keywords:** soil nematode community, management practice, *Bt* rice cultivation, insecticides application, paddy field

## Abstract

The wide-scale adoption of transgenic crops has aroused public concern towards potential impacts to the ecological services of soil fauna, such as soil nematodes. However, few studies has examined whether the cultivation of transgenic rice would pose greater threats to soil nematode community and associated ecological functions than insecticides application. Moreover, what are determinants of soil nematode community in paddy fields remains unclear. During a 3-year field study, rhizosphere soil samples of transgenic-Bt rice, its counterpart non-Bt parental rice and not-Bt rice with insecticides application were taken at four times in the rice developmental cycle using a random block design with three replications for each treatment. We hypothesized that the effects of pest management practice on soil nematode abundance and metabolic footprint change with trophic group and sampling time. We also predicted there were significant differences in structure and composition of soil nematode community across the three treatments examined and sampling times. In agreement with our expectation, the effects of pest management practice on nematode abundance and metabolic footprints depend on trophic group and sampling time. However, pest management practice exerted no apparent effect on nematode diversity and community composition. Soil nutrient availability and C:N molar ratio are the primary regulating factor of soil nematode community in rice paddy fields. In conclusion, our findings implied that changes in abundance, diversity, metabolic footprints associated with the crop growth stage overweighed the application of *Bt* rice and insecticides. The cultivation of *Bt* rice Huahui-1 exerted no measurable adverse effect on soil nematode community in rhizosphere soil over 3 years of rice cropping.

## Introduction

As an important cereal crop worldwide, rice (*Oryza sativa* L.) provides staple food and nutrition for about 50% of the global population (Lu and Snow, 2005). However, the yield of rice in China suffers severe losses mainly from four major lepidopteran pests, namely, the rice striped stem borer *Chilo suppressalis* (Crambidae), the yellow stem borer *Scirpophaga incertulas* (Crambidae), the pink stem borer *Sesamia inferens* (Noctuidae) and the rice leaf roller *Cnaphalocrocis medinalis* (Crambidae) (Chen et al., 2011). To reduce the yield loss resulting from pest damage, China devoted great effort in developing insect-resistant rice using transgenic technology and has developed multiple *Bt* rice lines (Chen et al., 2011). Empirical evidence supported *Bt* rice can result in an evidently decreased application of pesticides, and thus benefit human health and the environment (Huang et al., 2005; Chen et al., 2011). However, the *Cry1Ab/Ac* protein, which was continuously produced within plant tissue of *Bt*-rice, could be released through root exudates during growth and persistent in the rhizosphere in paddy soils (Wang et al., 2018). Therefore, it might affect the activity, structure, diversity of soil fauna, and interactions among components of soil food webs, which in turn would influence soil fertility and plant productivity. Accordingly, there is a pressing need to understand the impacts of *Bt* rice on the sustainability of agricultural ecosystem.

Nematodes play a pivotal role in ecosystem functions because they hold a central role in soil food webs and actively participate in ecological processes, such as decomposition, nutrient cycling, and pest suppression (Neher, 2010). Moreover, they react rapidly to disturbances and enrichment (Bongers, 1999). Therefore, the abundance and community composition of soil nematode have been commonly used to indicate soil health condition and soil functions (Bongers and Ferris, 1999; Neher, 2001). Ample available evidence support that soil nematodes are related to soil physicochemical properties (Briar et al., 2011; Godefroid et al., 2013), microorganisms (Kaplan and Noe, 1993), management practice (Biederman et al., 2008; Neher, 2010; Naira and Ngouajiob, 2012; Liu et al., 2016), and stochastic factors. The frequency of pesticide application in fields of *Bt* rice is lower than that of its counterpart non-Bt rice (Li et al., 2014). However, the *Cry1Ab/Ac* of *Bt* rice can enter soil ecosystems through root exudations, pollen and plant residue inputs, and thus nematodes are likely to be exposed to bioactive proteins from transgenic *Bt* rice because of their trophic position in soil food webs (Ruf et al., 2013). Moreover, the *Cry1Ab/Ac* proteins of Bt rice might accumulate in rhizosphere soil (Liu et al., 2018a). However, no definite conclusions have been drawn about whether *Bt* rice can negatively affect soil nematode. Besides, there are compounding factors affecting the persistence and accumulation of Bt-toxins, and the activity, persistence, and accumulation of *Bt* protein would vary with Bt toxin origin, plant species, and environment condition (Icoz and Stotzky, 2008; Chen et al., 2017a). For example, the activity and persistence of the insecticidal protein varies depending on climatic conditions (Zwahlen et al., 2003) and soil properties (Tapp et al., 1994; Saxena et al., 1999). Thus, whether *Bt* rice cultivation would affect soil nematodes and associated ecosystem functioning remains elusive, particularly under paddy field condition.

The primary objectives of this study were: 1) to quantify the effects of contrasting pest management practices on soil quality with soil nematode community as integrative bio-indicator in rice soil under paddy field condition; and 2) to explore the main driving force of soil nematode community in paddy fields. We also hypothesized that the effect size of *Bt* rice cultivation on soil nematode abundance and metabolic footprints changes with trophic group and plant developmental stage (exactly sampling time), because the interaction strength between plant and soil nematodes might vary with trophic group and the microclimate as well as the quantity and quality of plant detritus entering soils might vary with plant phenology. We also hypothesized that the impact of pesticides application on abundance, diversity, and footprints of soil nematode community would be greater than that of *Bt* rice cultivation, since pesticides are of broad spectrum whereas the toxicity of *Bt* rice is specific to target insects.

## Materials and Methods

### Site Description

A 3-year field experiment was carried out at the Jiangxi Academy of Agricultural Sciences, Jiangxi province (N28°21′91.4′′, E115°55′49.7′′), where the research of transgenic plants is permitted. The mean annual temperature and the mean annual rainfall of this region were 17.6°C and 1,624.4 mm, respectively. The soil here is loamy sand, and the physicochemical properties of the field soils were as follows: total soil organic carbon (SOC), 21.79 g/kg; total nitrogen (TN), 1.17 g/kg; total phosphorous (TP), 0.84 g/kg; total potassium (TK), 21.01 g/kg; available nitrogen (AN), 84.82 mg/kg; available phosphorous (AP), 5.82 mg/kg; and available potassium (AK), 49.17 mg/kg.

### Plant Materials

The transgenic *Bt* rice line (Huahui-1) and its corresponding non-transgenic counterparts (Minghui-63), which were provided by Huazhong Agricultural University, Wuhan, China were used in this experiment. The Huahui-1 is a certificate-granted insect-resistant rice line, it contains a fused *cry1Ab/1Ac* gene under the control of the rice actinI promoter showing a high level of expression of the δ-endotoxin, and thus is highly resistant to target insects under controlled conditions (Tu et al., 2000); while Minghui-63 is an elite Indica cytoplasmic male sterile restorer line developed in China in the early 1980s from the IR-30×Gui-630 hybrid combination.

### Experimental Design

The experiment was conducted using a randomized block design with three treatments including *Bt* rice, non-Bt with normal pesticide application, and non-Bt rice without pesticide application. For each treatment, there were three replications. Seeds were sown in mid-May, and seedlings were transplanted in mid-June in 2012, 2013, and 2014. The distance between rice seedlings was approximately 30 cm, which is commonly used in fields by farmers in this region. Rice was cultivated using standardized agricultural management practices except for pesticide application during the growing season. Weeds were controlled by hand-weeding every 3 weeks. The pests in *Bt* rice and one non-Bt fields were trapped by yellow sticky traps, whereas another non-Bt fields was controlled with insecticides spray.

### Soil Sampling and Analysis

Soil samples were collected on June 11, August 11, September 11, and October 11 of 2012, 2013, and 2014 at the seedling, booting, heading, and maturing stage of rice. We also took soil samples on April 11 of 2013, 2014, and 2015 corresponding to prophase of sowing. Before each sampling time, the flood irrigation was stopped for 3 days. In each plot, eight soil cores (2.5 cm in diameter) were collected randomly between rice rows, mixed thoroughly and pooled as a composite sample. The samples were placed in plastic bags and stored in a portable cooler for transport to the laboratory. Each soil sample was divided into two subsamples of equal volume. One was passed through a 2-mm sieve to remove root fragments and other organic debris in soil and stored at 4°C before testing. This subsample was used to determine soil water content (SWC) and soil nematode community. The other subsample was air-dried and sieved before using for the analysis of SOC and other soil properties including TN, TP, AN, AP, and AK.

Nematodes were extracted from 100 g of field-moisture soil from each subsample using the minor modified cotton-wool filter method depending on the nematode mobility (Liang et al., 2009) with three technical replicates for each sample within 1 week after sampling. After a 48 h of extraction, nematodes were killed through heating, and thus fixed and preserved in 4% formaldehyde. Subsequently, 10% of the individuals (but not less than 200 individuals, if possible) were additionally identified to genus level based on nematode morphology of the stoma, stylet, basal bulb, and teeth characteristics following Bongers (1994) and Ahmad and Jairajpuri (2010) at a 400× or 1,000× magnification. When the individual number of nematodes in a sample was less than 200, all the specimens were identified. All identified specimens were assigned into bacterial feeders, fungal feeders, plant feeders, omnivores and predators (Yeates et al., 1993), and c-p classes (Bongers and Bongers, 1998).

SOC content was measured using the hot oxidation with potassium dichromate and sulfuric acid (Yeomans and Bremner, 1988). Soil TN was determined by the semimicro-Kjeldahl method after soil was digested by HClO_4_ and HF. The contents of TP, TK, AK, and AP were assayed using inductively coupled plasma mass spectrometry (ICP-MS) analysis (IRIS Intrepid II XSP system; Thermo Electric Co., USA). The content of TP in soil was digested with H_2_SO_4_-HClO4 solution at 250°C and determined by the molybdenum-blue colorimetric method (Walker and Adams, 1958). The content of soil AP was extracted with 0.5 M NaHCO_3_ solution and determined by the molybdenum-blue colorimetric method (Olsen and Sommers, 1982).

### Statistical Analysis

The effect of pest management practice on abundance of soil nematodes was examined with generalized linear models, whereas that on diversity and metabolic footprints of soil nematode community was examined with general linear models. Subsequently, differences in composition of soil nematodes communities among treatments were investigated with non-metric multidimensional scaling (NMDS). Additionally, statistical differences in nematode community composition within plots of contrasting management practices and sampling times were assessed with “adonis” function based on 9,999 restricted permutations of the data. Finally, environmental variables which are related to the ordination of NMDS were selected with “envfit” function in vegan packages. The multivariate analyses including NMDS, adonis, and envfit, were performed with the “MASS” and the “vegan” package in R version 3.3.1 (R, 2016).

## Results

### Abundance of Soil Nematodes

In 2012 and 2013, pest management practice exerts significant effects on abundances of all trophic groups and total nematode abundance (**Table 1**). In 2014, pest management practice exerts significant effects on the abundances of bacterivores (Wald χ^2^ = 10.466, *P* = 0.005), omnivores (Wald χ^2^ = 35.613, *P* < 0.001), and total nematode abundance (Wald χ^2^ = 15.847, *P* < 0.001), whereas negligible effect on abundances of other trophic groups (**Table 1**). Specifically, the pesticide application greatly reduced nematode abundance, whereas *Bt* rice did not. Additionally, nematode abundances of the non-Bt rice fields with pesticides application were significantly lower in comparison with those of *Bt* rice fields. However, there were significant interactive effects of management *sampling time, suggesting the effect of pest management practice on nematode abundance depended on sampling time.

**Table 1:** Summary of generalized linear models testing the effects of management practice, sampling time, and their interaction on abundance of soil nematodes in rice fields of contrasting management practices during 2012–2014 growing seasons.

Year	Variable	Parameter	Management	Sampling time	Interaction
	All	*df*	2	3	6
2012	Herbivores	Waldχ^2^	52.651	1,280.983	31.362
		*P*	**<0.001**	**<0.001**	**<0.001**
	Bacterivores	Waldχ^2^	5.487	579.862	98.021
		*P*	**< 0.0064**	**<0.001**	**<0.001**
	Fungivores	Waldχ^2^	17.351	311.93	60.462
		*P*	**<0.001**	**<0.001**	**<0.001**
	Omnivores	Waldχ^2^	6.657	16.856	33.12
		*P*	**0.036**	**0.001**	**<0.001**
	Predators	Waldχ^2^	33.941	143.775	105.445
		*P*	**<0.001**	**<0.001**	**<0.001**
	Total	Waldχ^2^	139.497	1,262.296	96.973
		*P*	**<0.001**	**<0.001**	**<0.001**
2013	Herbivores	Waldχ^2^	95.563	254.174	25.206
		*P*	**<0.001**	**<0.001**	**<0.001**
	Bacterivores	Waldχ^2^	93.906	509.785	106.725
		*P*	**<0.001**	**<0.001**	**<0.001**
	Fungivores	Waldχ^2^	169.109	250.999	189.805
		*P*	**<0.001**	**<0.001**	**<0.001**
	Omnivores	Waldχ^2^	115.414	483.906	19.799
		*P*	**<0.001**	**<0.001**	**0.003**
	Predators	Waldχ^2^	339.198	1,176.536	51.586
		*P*	**<0.001**	**<0.001**	**<0.001**
	Total	Waldχ^2^	824.096	1,215.524	109.763
		*P*	**<0.001**	**<0.001**	**<0.001**
2014	Herbivores	Waldχ^2^	0.527	400.864	68.868
		*P*	0.768	**<0.001**	**<0.001**
	Bacterivores	Waldχ^2^	10.466	274.138	21.468
		*P*	**0.005**	**<0.001**	**0.002**
	Fungivores	Waldχ^2^	5.156	785.646	15.91
		*P*	0.076	**<0.001**	**<0.001**
	Omnivores	Waldχ^2^	35.613	848.327	49.619
		*P*	**<0.001**	**<0.001**	**<0.001**
	Predators	Waldχ^2^	2.541	297.212	86.6.
		*P*	0.281	**<0.001**	**<0.001**
	Total	Waldχ^2^	15.847	1,668.931	155.559
		*P*	**<0.001**	**<0.001**	**<0.001**

The bolded results indicate difference in variables across treatments are statistically significant (p value < 0.05).

### Diversity of Soil Nematode Community

Pest management practice was found be of no significant effect on the taxa richness, Margalef richness index, Shannon–Weaver diversity index, Simpson dominance index, and Pielou evenness index of soil nematode community (**Table 2**). The diversity indices vary greatly across sampling times, but they are independent of management practice.

**Table 2:** Summary of general linear models testing the effects of management practice, sampling time, and their interaction on the diversity of soil nematode community in rice fields during 2012–2014 growing seasons.

Year	Variable	Management practice			Sampling time			Interaction		
		*df*	*F*	*P*	*df*	*F*	*P*	*df*	*F*	*P*
2012	S	2	1.353	0.277	3	8.885	**<0.001**	6	0.480	0.817
	SR	2	0.426	0.658	3	5.338	**0.006**	6	0.289	0.937
	H'	2	0.284	0.755	3	4.494	**0.012**	6	1.166	0.357
	λ	2	0.992	0.386	3	4.018	**0.019**	6	1.158	0.361
	J	2	0.885	0.426	3	2.566	0.078	6	1.074	0.405
2013	S	2	0.553	0.582	3	4.573	**0.011**	6	0.482	0.815
	SR	2	1.662	0.211	3	2.539	0.080	6	0.789	0.588
	H'	2	0.386	0.684	3	4.409	**0.013**	6	0.386	0.881
	λ	2	0.340	0.715	3	2.758	0.064	6	0.435	0.848
	J	2	0.925	0.410	3	3.348	**0.036**	6	0.665	0.679
2014	S	2	0.051	0.951	3	28.675	**<0.001**	6	0.624	0.709
	SR	2	0.107	0.899	3	13.517	**<0.001**	6	0.568	0.751
	H'	2	0.019	0.982	3	18.867	**<0.001**	6	0.418	0.860
	λ	2	0.096	0.909	3	14.538	**<0.001**	6	0.498	0.804
	J	2	0.032	0.969	3	13.684	**<0.001**	6	0.65	0.69

S, taxa richness; SR, Margalef richness index; H', Shannon–Weaver diversity index; λ, Simpson dominance index; J, Pielou evenness index.

The bolded results indicate difference in variables across treatments are statistically significant (p value < 0.05).

### Metabolic Footprints of Soil Nematodes

The effects of management practice on metabolic footprints of soil nematodes vary depending on year and trophic group examined. In 2012, no detectable difference in metabolic footprints examined among contrasting management practices of rice was observed. In 2013, herbivore footprint and predator footprint changed with management practice. In 2014, management practice exerted a significant effect on composite footprint, structure footprint, bacterivore footprint and omnivore footprint of soil nematode community. Additionally, metabolic footprints of soil nematodes varied with sampling time in most cases. However, the effects of management practice on metabolic footprints did not change with sampling time in most cases (**Table 3**).

**Table 3:** Summary of general linear models testing the effects of management practice, sampling time and their interaction on the metabolic footprints of soil nematode community in rice fields during 2012–2014 growing seasons.

Year	Variable	Management practice			Sampling time			Interaction		
		*df*	*F*	*P*	*df*	*F*	*P*	*df*	*F*	*P*
2012	CMF	2	0.598	0.558	3	2.014	0.139	6	0.176	0.981
	EMF	2	0.666	0.523	3	13.665	**<0.001**	6	0.315	0.923
	SMF	2	0.392	0.68	3	0.779	0.517	6	0.18	0.98
	HMF	2	1.858	0.178	3	28.639	**<0.001**	6	0.885	0.521
	FMF	2	0.363	0.699	3	4.642	**0.011**	6	0.87	0.531
	BMF	2	0.653	0.53	3	9.148	**<0.001**	6	0.221	0.966
	PMF	2	2.051	0.151	3	11.165	**<0.001**	6	1.350	0.274
	OMF	2	0.451	0.642	3	2.205	0.114	6	0.041	1.00
2013	CMF	2	2.479	0.105	3	5.797	**0.004**	6	0.224	0.965
	EMF	2	2.010	0.156	3	2.856	0.058	6	1.099	0.392
	SMF	2	2.381	0.114	3	6.301	**0.003**	6	0.236	0.96
	HMF	2	4.366	**0.024**	3	7.869	**0.001**	6	0.550	0.765
	FMF	2	3.004	0.069	3	2.940	**0.054**	6	1.098	0.392
	BMF	2	1.948	0.164	3	3.612	**0.028**	6	0.894	0.515
	PMF	2	3.864	**0.035**	3	7.91	**0.001**	6	0.684	0.665
	OMF	2	1.471	0.25	3	4.82	**0.009**	6	0.113	0.994
2014	CMF	2	13.256	**<0.001**	3	64.812	**<0.001**	6	9.164	**<0.001**
	EMF	2	2.86	0.077	3	85.813	**<0.001**	6	1.985	0.108
	SMF	2	13.192	**<0.001**	3	61.778	**<0.001**	6	9.174	**<0.001**
	HMF	2	0.604	0.555	3	44.736	**<0.001**	6	3.755	**0.009**
	FMF	2	0.563	0.577	3	113.882	**<0.001**	6	0.676	0.67
	BMF	2	4.026	**0.031**	3	43.949	**<0.001**	6	4.254	**0.005**
	PMF	2	0.465	0.634	3	10.072	**<0.001**	6	1.442	0.24
	OMF	2	21.684	**<0.001**	3	106.575	**<0.001**	6	13.422	**<0.001**

BMF, bacterivore footprint; CMF, composite footprint; EMF, enrichment footprint; FMF, fungivore footprint; HMF, herbivore footprint; OMF, omnivore footprint; PMF, predator footprint; SMF, structure footprint. The bolded results indicate difference in variables across treatments are statistically significant (p value < 0.05).

### Determinant of Soil Nematode Community in Paddy Fields

**Figure 1:**
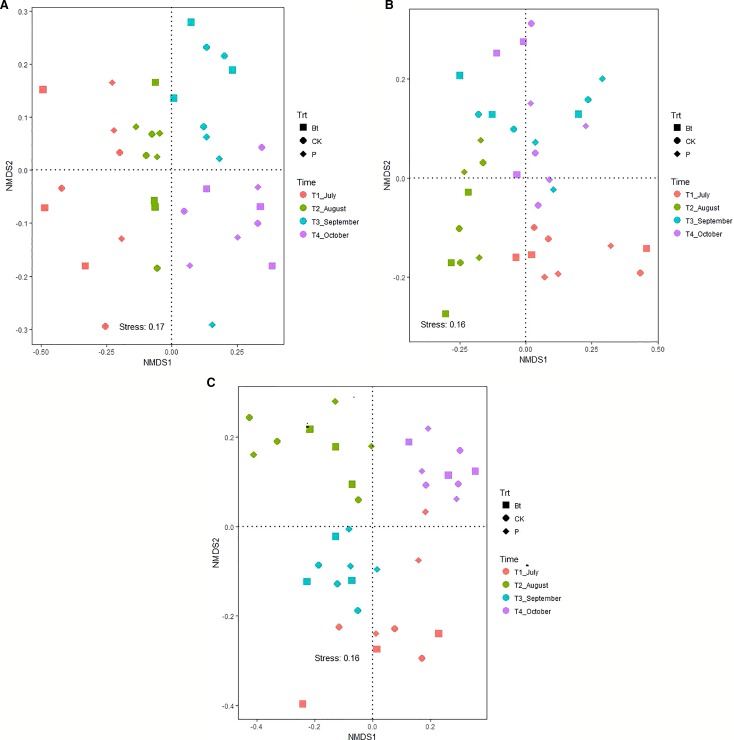
Non-metric multidimensional scaling (NMDS) illustrating the divergence in community composition of soil nematodes in soils of rice fields under different mangement practices in 2012 **(A)**, 2013 **(B)**, and 2014 **(C)**. The plot of NMDS was produced using the Bray-Curtis distance. CK: non-Bt rice without pesticide application; Bt: Bt rice without pesticide application; P: non-Bt rice with pesticide application.

**Figure 2:**
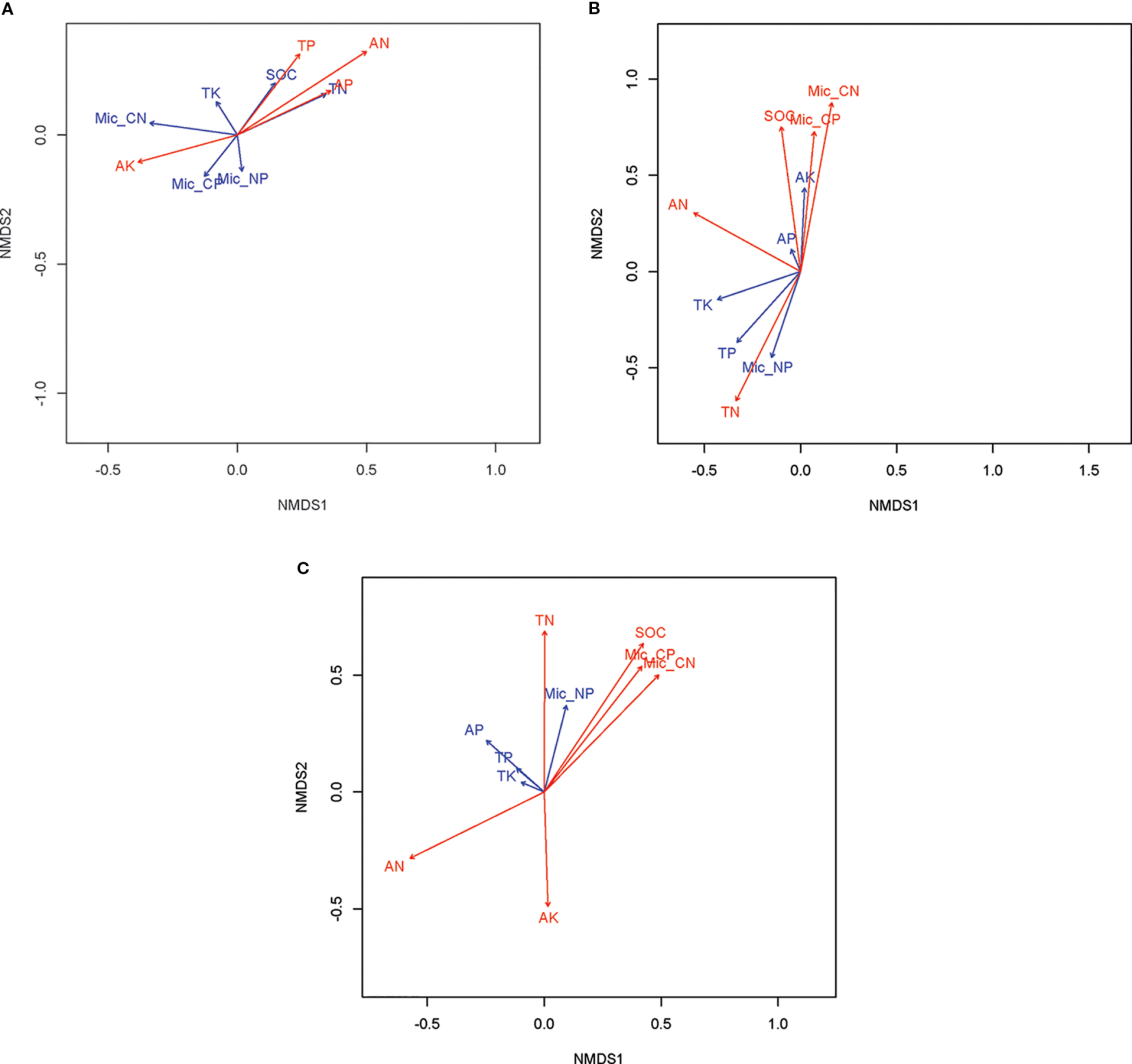
Non-metric multidimensional scaling (NMDS) revealing the major shaping factors of soil nematode community composition in soils of rice fields under different mangement practices in 2012 **(A)**, 2013 **(B)**, and 2014 **(C)**. The plot of NMDS was produced using the Bray-Curtis distance e. The red vectors show the correlation between soil attributes and soil nematode community composition are significant at *P* < 0.05.

The nematode taxa richness in soils of rice paddy fields in the 2012, 2013 and 2014 growing season was 25, 21, and 22, respectively (**Appendix A**). With exception of the 2013 growing season, sampling date (*P < 0.05*) rather than management practice (*P > 0.05*) affect community composition of soil nematodes (**Table 4**; **Figure 1**). In 2012, the community composition of soil nematodes in rice fields correlate with available nutrients (including available N, P, and K), TN and TP in soils (**Figure 2A**). In 2013, the community composition of soil nematodes in rice fields correlate with available N, SOC, TN, TP, and C:N molar ratio in soils and soil microbial biomass (**Figure 2B**). In 2014, the community composition of soil nematodes in rice fields correlate with available N, available K, SOC, TN, TP, and C:N molar ratio in soils and soil microbial biomass (**Figure 2C**).

**Table 4:** Similarity of soil nematode community in Bt rice field without insecticide application, non-Bt rice fields with and without insecticide application.

Year	Source	*df*	*F*	*P*
2012	Management practice	2	1.02	0.399
	Sampling time	3	10.86	**0.001**
	Interaction	6	0.76	0.784
2013	Management practice	2	2.13	**0.042**
	Sampling time	3	4.93	**0.001**
	Interaction	6	0.48	0.988
2014	Management practice	2	1.53	0.168
	Sampling time	3	25.86	**0.001**
	Interaction	6	1.7	**0.053**

The bolded results indicate difference in variables across treatments are statistically significant (p value < 0.05).

## Discussion

The wide-scale adoption of *Bt* crops evidently reduces chemical insecticides application (Huang et al., 2005; Lu et al., 2012). However, *Bt* crops pose potential environmental risk to soil biota because they might change the quantity and quality of nutrient inputs and directly impact soil organisms with toxic activity of *Bt* protein. To date, whether the positive effects of the cultivation of *Bt* crop due to decreased application of chemical insecticides may outweigh its potential negative effects is unclear. Moreover, the responses of soil nematode to certain *Bt* transformation event was found to be context-specific, and thus a case-by-case study is necessary. China has developed multiple *Bt* rice lines to control target lepidopteran pests and boost agricultural productivity. For example, the Huahui-1 has been granted bio-safety certificates by the Chinese authorities since 2009. However, it has not been allowed to enter the Chinese agricultural system due to public concern towards its potential adverse environmental effects and food safety. Therefore, to enhance the communication about science related issues of *Bt* rice to public maybe a promising alternative of the commercial production of *Bt* rice in near future. The present study utilized the scare opportunity to figure out the potential environmental risks of the Huahui-1 on agricultural ecosystem.

### No Apparent Impact of *Bt* Rice Cultivation on Soil Nematodes

In contrast to most of available studies addressing potential environment risks of *Bt* corn or cotton on soil nematode community in terrestrial ecosystems (Manachini and Lozzia, 2002; Griffiths et al., 2005; Höss et al., 2011; Karuri et al., 2013; Li and Liu, 2013; Neher et al., 2014; Yang et al., 2014; Höss et al., 2015; Liu et al., 2015; Čerevková et al., 2017; Liu et al., 2018b), we evaluated those of *Bt* rice in aquatic ecosystem. Our study revealed no significant adverse effect of *Bt* rice cultivation on soil nematode communities under paddy field. Our results are in agreement with other studies suggesting that no impact of *Bt* crops cultivation on either soil nematodes (Manachini and Lozzia, 2002; Griffiths et al., 2005; Höss et al., 2011; Karuri et al., 2013; Li and Liu, 2013; Neher et al., 2014; Yang et al., 2014; Höss et al., 2015; Liu et al., 2015; Chen et al., 2017a; Chen et al., 2017b; Liu et al., 2018b) or other aquatic fauna (Wang et al., 2013; Li et al., 2014; Wang et al., 2014). One possible interpretation is that the concentration of *Cry* protein in field soils is lower than the threshold value which is adverse to the reproduction and growth rate of soil nematodes. There are several reasons for the low *Cry* protein concentration in the rice paddy soil. On one hand, the concentration of *Cry* proteins that enter soils is relatively lower or it is diluted in water. On the other hand, *Cry* proteins may be readily degraded (Wang et al., 2007; Liu et al., 2018b). In a study with the same rice cultivar (Huahui-1), the authors suggest that *Cry1Ab/Ac* proteins could not be detected in irrigation water of the rhizotrons (Liu et al., 2018a).

### More Noticeable Change in Soil Nematodes Community With Time in Comparison With Application of Pesticides and *Bt* Rice

In contrast to most of available studies, which only focused on the difference in parameters of soil nematode community between *Bt* and non-Bt corn or cotton (Manachini and Lozzia, 2002; Griffiths et al., 2005; Höss et al., 2011; Karuri et al., 2013; Li and Liu, 2013; Neher et al., 2014; Yang et al., 2014; Höss et al., 2015; Liu et al., 2015; Čerevková et al., 2017; Chen et al., 2017a; Chen et al., 2017b), we set up another treatment that is the not-Bt crop field with chemical pesticides application when necessary. This is necessary because non-Bt crop refuge has been proposed as a promising strategy for delaying the resistance development towards *Bt* crop (Gould, 2000; Tabashnik and Carriere, 2017). However, previous studies reported that planthoppers move from *Bt* to adjacent non-Bt rice fields (Chen et al. 2003a; Wang et al., 2018) and that fungal diseases and non-target pests still required to be controlled with pesticides application when full yield is expected to be achieved (Wang et al., 2010). This indicates even if the cultivation of *Bt* rice cultivation has a great potential to reduce the use of broad-spectrum chemical insecticides (High et al., 2004), pesticides application in *Bt* rice is still required. However, pesticide sprays is neither effective nor environment-friendly (Frutos et al., 1999). For example, one study demonstrates that pesticide application results in biodiversity loss in rice-based ecosystems (Halwart, 2008). Another studies reported that pesticide (acetochlor) application at a high dose could reduce plant parasites (Waliyar et al., 1992), the *Helicotylenchus* (Todd et al., 1992), the *Pratylenchus* in soil (Shukla and Haseeb, 1996; Zhang et al., 2010). Chen et al. (2003a) indicated that pesticide (acetochlor) impacted the numbers of total nematode and trophic groups in a Chinese soybean field. Pen-Mouratov and Steinberger (2005) found pesticides application reduced the numbers of total nematodes, fungivores, and bacterivores in a desert system. Wada and Toyota (2008) reported pesticide effectively suppressed a *Pratylenchus penetrans*, but had little impact on free-living nematodes in a pot test. The discrepancy across studies may relate to pesticide type, application dose, and sensitivity of nematode species. Our findings highlight that the effects of year and sampling date were more pronounced than that of application of pesticide and Bt rice.

### Factors Shaping Soil Nematode Community in Rice Fields Under Paddy Condition

Against our expectation and most studies that reporting agricultural management practice greatly impact soil nematode community (Sánchez-Moreno et al., 2009; Palomares-Rius et al., 2012), agricultural management did not change nematode community composition in the present study (**Table 4**). In agreement with the finding of similar issue focusing on plant parasites in Spain olive fields (Archidona-Yuste et al., 2020), soil was the following most influential factor driving nematode communities in rice paddy fields. Numerous studies suggest that nematode was closely related to soil physiochemical factors (Ferris et al., 1996; Wardle et al., 2004; Archidona-Yuste et al., 2020). For example, SOC and TN were found to be significantly associated with total nematodes abundance (Ma et al., 2018). In the present study, available N, SOC, and TN are important factors shaping soil nematode community in rice fields under paddy condition (**Figure 2**). Concerning the found close relationship between N and nematode, a reasonable explanation is that nematodes affect nitrogen availability both directly and indirectly (Ingham et al., 1985; Neher, 2010). Regarding the observed close correlation between soil nematode community and soil C/N and microbial biomass, it is not surprisingly. After all, C/N is a promising soil quality indicator, and it has been reported as an important influencing factor of terrestrial nematode biodiversity (Mulder and Maas, 2017).

## Conclusion

In summary, our results give the public the actual environmental risk of *Bt* rice cultivation to soil nematodes and their associated ecological functions. All the findings support that there is no deleterious effect of *Bt* rice cultivation on soil nematode community over 3 years. Additionally, the effects of year and sampling date on nematode variables examined in rhizosphere soil were more pronounced than that of application of insecticides and *Bt* rice. Soil nutrient availability and C:N molar ratio are the primary regulating factor of soil nematode community in rhizosphere soil of rice under paddy field condition.

## Data Availability Statement

The datasets generated for this study are available on request to the corresponding authors.

## Author Contributions

BY, FC and FG designed the experiment. QC, XL, YL and FC did the experiment. BY, WQ, and LH analyzed the data. All the authors wrote and improved the manuscript.

## Funding

This study was jointly supported by the National Natural Science Foundation of China (Grant number: 31400360), the State Scholarship Fund of the Chinese Scholarship Council (grant number: 201604910484), and the Special Program for New Transgenic Variety Breeding of the Ministry of Science and Technology, China (2016ZX08012005). The authors thank professor Carolina Escobar and two anonymous reviewers for their critical and constructive suggestions of an earlier draft.

## Conflict of Interest

The authors declare that the research was conducted in the absence of any commercial or financial relationships that could be construed as a potential conflict of interest.
