# Supplementary material for: Effects of Pest Management Practices on Soil Nematode Abundance, Diversity, Metabolic Footprint and Community Composition Under Paddy Rice Fields
Source: Front Plant Sci. 2020 Feb 19;11:88. doi: 10.3389/fpls.2020.00088 (PMC7042464; doi:10.3389/fpls.2020.00088)
Supplement: Supplementary file 1 [file DataSheet_1.docx]

**APPENDIX A** Nematode taxa found in the study. The classification of feeding groups and c-p scores were according to Yeates *et al*. (1993) and Bongers and Bongers (1998)

| Year | Taxa | c-p scores | Trophic groups | |
| --- | --- | --- | --- | --- |
| 2012 | *Acrobeloides* | 2 | Bacterivores | |
|  | *Actinolaimus* | 4 | Omnivores | |
|  | *Alaimus* | 4 | Bacterivores | |
|  | *Aphelenchoides* | 2 | Fungivores | |
|  | *Aphelenchus* | 2 | Fungivores | |
|  | *Aporcelaimus* | 5 | Omnivores | |
|  | *Chronogaster* | 3 | Bacterivores | |
|  | *Criconema* | 3 | Herbivores - ectoparasites | |
|  | *Dorylaimus* | 4 | Omnivores | |
|  | *Ethmolaimus* | 3 | Bacterivores | |
|  | *Eucephalobus* | 2 | Bacterivores | |
|  | *Filenchus* | 2 | Fungivores | |
|  | *Helicotylenchus* | 3 | Herbivores - semi-endoparasites | |
|  | *Hirschmanniella* | 3 | Herbivores - migratory endoparasites | |
|  | *Mesorhabditis* | 1 | Bacterivores | |
|  | *Mononchus* | 4 | Predators |  |
|  | *Nothotylenchus* | 2 | Fungivores | |
|  | *Nygolaimus* | 5 | Predators |  |
|  | *Panagrolaimus* | 1 | Bacterivores | |
|  | *Pratylenchus* | 3 | Herbivores - migratory endoparasites | |
|  | *Prismatolaimus* | 3 | Bacterivores | |
|  | *Prodorylaimus* | 4 | Omnivores | |
|  | *Tobrilus* | 3 | Predators |  |
|  | *Tripyla* | 3 | Predators |  |
|  | *Tylenchorhynchus* | 3 | Herbivores - ectoparasites | |
| 2013 | *Actinolaimus* | 4 | Omnivores | |
|  | *Alaimus* | 4 | Bacterivores | |
|  | *Aphelenchoides* | 2 | Fungivores | |
|  | *Aporcelaimus* | 5 | Omnivores | |
|  | *Chronogaster* | 3 | Bacterivores | |
|  | *Dorylaimus* | 4 | Omnivores | |
|  | *Ethmolaimus* | 3 | Bacterivores | |
|  | *Eucephalobus* | 2 | Bacterivores | |
|  | *Filenchus* | 2 | Fungivores | |
|  | *Hirschmanniella* | 3 | Herbivores - migratory endoparasites | |
|  | *Mesodorylaimus* | 4 | Omnivores | |
|  | *Mesorhabditis* | 1 | Bacterivores | |
|  | *Mononchus* | 4 | Predators |  |
|  | *Panagrolaimus* | 1 | Bacterivores | |
|  | *Plectus* | 2 | Bacterivores | |
|  | *Pratylenchus* | 3 | Herbivores - migratory endoparasites | |
|  | *Prodorylaimus* | 4 | Omnivores | |
|  | *Thornia* | 4 | Omnivores | |
|  | *Tobrilus* | 3 | Predators |  |
|  | *Tripyla* | 3 | Predators |  |
|  | *Tylenchorhynchus* | 3 | Herbivores - ectoparasites | |
| 2014 | *Actinolaimus* | 4 | Omnivores | |
|  | *Alaimus* | 4 | Bacterivores | |
|  | *Aphelenchoides* | 2 | Fungivores | |
|  | *Aporcelaimus* | 5 | Omnivores | |
|  | *Chronogaster* | 3 | Bacterivores | |
|  | *Diploscapter* | 1 | Bacterivores | |
|  | *Dorylaimus* | 4 | Omnivores | |
|  | *Epidorylaimus* | 4 | Omnivores | |
|  | *Ethmolaimus* | 3 | Bacterivores | |
|  | *Eucephalobus* | 2 | Bacterivores | |
|  | *Filenchus* | 2 | Fungivores | |
|  | *Helicotylenchus* | 3 | Herbivores - semi-endoparasites | |
|  | *Hirschmanniella* | 3 | Herbivores - migratory endoparasites | |
|  | *Mesodorylaimus* | 4 | Omnivores | |
|  | *Mononchus* | 4 | Predators |  |
|  | *Nothotylenchus* | 2 | Fungivores | |
|  | *Panagrolaimus* | 1 | Bacterivores | |
|  | *Prodorylaimus* | 4 | Omnivores | |
|  | *Thornia* | 4 | Omnivores | |
|  | *Tobrilus* | 3 | Predators |  |
|  | *Tripyla* | 3 | Predators |  |
|  | *Tylenchorhynchus* | 3 | Herbivores - ectoparasites | |
